# Psychometric Properties of the Difficulties in Emotion Regulation Scale (DERS) and Its Short Forms in Adults With Emotional Disorders

**DOI:** 10.3389/fpsyg.2018.00539

**Published:** 2018-04-19

**Authors:** Lauren S. Hallion, Shari A. Steinman, David F. Tolin, Gretchen J. Diefenbach

**Affiliations:** ^1^Anxiety Disorders Center, Center for Cognitive Behavioral Therapy, Institute of Living, Hartford Hospital, Hartford, CT, United States; ^2^Department of Psychology, University of Pittsburgh, Pittsburgh, PA, United States; ^3^Department of Psychology, West Virginia University, Morgantown, WV, United States; ^4^Yale School of Medicine, Yale University, New Haven, CT, United States

**Keywords:** emotion regulation, difficulties in emotion regulation scale, emotional disorders, treatment outcome, psychometrics, structural equation modeling, cognitive-behavioral therapy

## Abstract

**Objective:** The Difficulties in Emotion Regulation Scale (DERS) is a widely used self-report measure of subjective emotion ability, as defined by a prominent clinically derived model of emotion regulation (Gratz and Roemer, 2004). Although the DERS is often used in treatment and research settings for adults with emotional (i.e., anxiety, mood, obsessive-compulsive, or trauma-related) disorders, its psychometric properties are not well-characterized in this population.

**Method:** We examined the psychometric properties of the DERS and three popular short forms (DERS-16; DERS-18; and DERS-SF) in a large (*N* = 427) sample of treatment-seeking adults with one or more *DSM-5* emotional disorders.

**Results:** For the original DERS, internal consistency was strong for all subscales except Awareness. A bifactor structure consisting of one general emotion dysregulation factor and five uncorrelated specific factors corresponding to the original DERS subscales (excluding Awareness) provided the best fit. A series of structural equation models (SEMs) demonstrated unique incremental contributions of the general factor and several specific factors to explaining concurrent clinical severity. The general factor and one specific factor (Goals) also prospectively predicted treatment outcome following a naturalistic course of outpatient cognitive-behavioral therapy (CBT) in a subset of participants (*n* = 202) for whom discharge data were available. Specifically, more severe emotion dysregulation at intake predicted better CBT response, while more severe impairment in goal-directed activity when distressed predicted worse CBT response. All three short forms showed a robust bifactor structure and good internal consistency and convergent validity vis-à-vis the original measure, albeit with a slight decrement in incremental utility (1–3% less variance explained in clinical severity).

**Conclusion:** With the Awareness items excluded, the DERS showed good internal consistency and a robust bifactor latent structure. The general factor and several specific factors incrementally and prospectively predicted clinical severity and treatment outcome, which suggests that the DERS may have clinical and predictive utility in treatment-seeking adults with emotional disorders. Additional research is needed to establish convergent and discriminant validity in this population. The use of a short form in lieu of the full DERS may be sufficient for many general clinical and research purposes, particularly when participant burden is a concern.

## Introduction

The Difficulties in Emotion Regulation Scale (DERS; Gratz and Roemer, 2004) is a popular but controversial self-report measure that aims to assess emotion dysregulation, broadly conceptualized. The original validation paper has been cited nearly 3,000 times to date, has been translated into several languages, and has spurred the development of several short forms (e.g., DERS-16, Bjureberg et al., 2016; DERS-SF, Kaufman et al., 2016; and DERS-18, Victor and Klonsky, 2016). The theoretical model from which the DERS is derived (Gratz and Roemer, 2004) has its roots in “third-wave” models of cognitive behavioral therapy, which propose a central role for experiential avoidance in the onset and maintenance of most forms of emotional disturbance. Experiential avoidance is defined as intolerance of and maladaptive efforts to avoid (usually negative) emotional experiences (e.g., Hayes et al., 1996). Within this framework, emotion regulation abilities are viewed as intact when the individual is able to behave in a way that facilitates the achievement of *a priori* goals, particularly in the context of negative affect or other strong emotional experiences.

The model upon which the DERS is based (Gratz and Roemer, 2004) proposes four broad facets of emotion regulation: (a) awareness and understanding of emotions; (b) acceptance of emotions; (c) the ability to control impulses and behave in accordance with goals in the presence of negative affect; and (d) access to emotion regulation strategies that are perceived to be effective for feeling better. This model has been embraced primarily within applied clinical research and treatment contexts. Critically, this clinical-contextual model of emotion regulation is entirely distinct from leading models of emotion regulation derived from basic affective science (e.g., Gross, 1998; Aldao, 2013; Gross and Jazaieri, 2014). Affective science-based frameworks tend to conceptualize emotion regulation more narrowly and tend to focus more on process than on presumed trait-level abilities (Gross, 1998, 2015; Aldao, 2013; Gross and Jazaieri, 2014). As such, the extent to which any measure derived from a clinical-contextual framework could be considered a measure of emotion regulation as it is defined by affective scientists is a matter of debate. The present paper does not attempt to resolve this controversy. Rather, the present study is contextualized within the agnostic observation that although DERS is widely used in treatment and research settings for adults with emotional (i.e., *DSM-5* anxiety, depressive, bipolar, obsessive-compulsive, and trauma- and stressor-related) disorders, its psychometric properties have not yet been adequately characterized in this population.

The DERS was designed to assess trait-level perceived emotion regulation ability as defined by the Gratz and Roemer (2004) clinical-contextual framework. The measure is scored such that higher scores reflect greater impairment or dysregulation. Exploratory factor analysis (EFA) in the original development and validation study suggested a six- or seven-factor structure. The six-factor structure was deemed more interpretable and was translated into six subscales: (a) lack of emotional awareness (*Awareness*; “I am attentive to my feelings,” reverse-scored); (b) lack of emotional clarity (*Clarity*; “I have difficulty making sense out of my feelings”); (c) difficulty regulating behavior when distressed (*Impulse*; “When I’m upset, I become out of control”); (d) difficulty engaging in goal-directed cognition and behavior when distressed (*Goals*; “When I’m upset, I have difficulty getting work done”); (e) unwillingness to accept certain emotional responses (*Non-acceptance*; “When I’m upset, I become angry at myself for feeling that way); and (f) lack of access to strategies for feeling better when distressed (*Strategies*; “When I’m upset, I believe there is nothing I can do to feel better”).

Several subsequent factor analytic studies provide support for the original six-factor model provides an adequate fit in a variety of populations, including undergraduate students (Perez et al., 2012) and adolescents (Weinberg and Klonsky, 2009; Neumann et al., 2010). However, a number of studies have found poor fit for a six-factor solution in a variety of populations, including Italian undergraduate students (Giromini et al., 2012), chronic pain patients (Kökönyei et al., 2014), adults with severe mental illness (Fowler et al., 2014), and adult outpatients receiving Dialectical Behavior Therapy (DBT; Osborne et al., 2017). These studies generally find that a revised five-factor model that excludes the Awareness subscale and items provides a better fit to the data (Bardeen et al., 2012; Fowler et al., 2014; Osborne et al., 2017).

Notably, studies that have attempted to fit a higher-order factor in addition to the six lower-order factors have generally found relatively poor fit, although fit is again improved when the Awareness items are excluded (Bardeen et al., 2012; Fowler et al., 2014). These findings were conceptually replicated in a study that attempted to address possible psychometric problems related to reverse-scored items by rewording the items prior to administration (Bardeen et al., 2016). Notably, one recent study (Osborne et al., 2017) provided preliminary support for a bifactor solution in a sample of *N* = 344 adults receiving DBT. A bifactor model typically includes one general factor that accounts for common variance across the items as well as one or more specific lower-order factors (e.g., Reise et al., 2010). These specific factors are not permitted to correlate with the general factor or each other and therefore are proposed to represent a latent construct that is unique and incremental relative to the general factor and the other specific factors. Support for a bifactor model would suggest that the DERS may indeed assess five (or six) distinct but related latent constructs. However, the replicability of this finding and its generalizability to other clinical populations has not been assessed, nor has the extent to which any specific factors incrementally predict relevant clinical variables above variance explained by the general factor. The present paper aims to address both gaps in the literature.

A substantial body of research has shown significant positive associations between scores on the DERS (specifically the total score) and symptoms of a range of psychological disorders, including borderline personality disorder (Gratz et al., 2006), generalized anxiety disorder (Mennin et al., 2002), substance use disorders (Fox et al., 2007; Gratz and Tull, 2010), social anxiety (Rusch et al., 2012), health anxiety (Bardeen and Fergus, 2014), post-traumatic stress disorder (Ehring and Quack, 2010), and bipolar disorder (Becerra et al., 2013; Van Rheenen et al., 2015). These findings provide preliminary evidence for the construct validity of the measure within the Gratz and Roemer (2004) framework and are broadly consistent with theoretical models that highlight emotion dysregulation as a transdiagnostic vulnerability factor for emotional disorders (e.g., Gross and Muñoz, 1995).

Findings with respect to specific subscale scores are less consistent than those for the total score. Perhaps the most consistent finding is that, consistent with the factor analytic results, the Awareness subscale tends to perform poorly, as evidenced by weak or absent associations with the other subscales and many indices of psychopathology (e.g., Tull et al., 2007, 2010; McDermott et al., 2009; Bardeen and Fergus, 2014). Consequently, an increasing number of studies and at least one short form (Bjureberg et al., 2016; DERS-16) exclude this subscale and its items from analysis.

Aside from the Awareness subscale, several studies in unselected and undergraduate samples have found positive associations for all or nearly all DERS subscales with various forms of anxiety (e.g., Salters-Pedneault et al., 2006; Tull et al., 2007) and other symptoms (e.g., Becerra et al., 2013). Other studies find that a single subscale, often the Strategies subscale, or just a few subscales best predict symptoms after the other subscales controlled (e.g., Rusch et al., 2012; Bardeen and Fergus, 2014). Fewer studies have examined the incremental utility of the subscales in clinical samples. In a sample of *N* = 218 adolescent inpatients (Perez et al., 2012), only the Strategies subscale accounted for a significant portion of the variance in non-suicidal self-injury (NSSI) after other facets of emotion dysregulation and broad diagnostic status (internalizing vs. externalizing disorder) were controlled. In a study of *N* = 60 participants with pure or comorbid obsessive-compulsive disorder (OCD) and hoarding disorder (de la Cruz et al., 2013), small to moderate zero-order associations were found between the Goals, Impulse, and Strategies subscales and several self-report and clinician-rated symptom severity measures; however, incremental utility was not examined. In a sample of 50 adults with bipolar disorder, the Strategies subscale was uniquely (incrementally) associated with depression severity after comorbidity and the other subscales were controlled.

The present study examined the psychometric properties of the DERS in a large (*N* = 427) transdiagnostic sample of treatment-seeking adults with emotional disorders. We were interested in the internal consistency and factor structure of the DERS, as well as the incremental utility of the various subscales for predicting clinical severity. We also aimed to provide a preliminary evaluation of the predictive validity of the DERS, including whether and to what extent the DERS and its subscales could predict response to cognitive-behavioral treatment (CBT). Finally, we briefly examined the psychometric properties of three recently published short forms of the DERS (e.g., DERS-16, Bjureberg et al., 2016; DERS-SF, Kaufman et al., 2016; and DERS-18, Victor and Klonsky, 2016) to evaluate the extent to which these measures performed comparably to the original measure. These analyses were undertaken because short forms reduce participant burden and can therefore be useful research and clinical tools if they are determined to be psychometrically sound. Specifically, we examined the internal consistency of the short form subscales, their convergent validity and factor structure vis-à-vis the original measure, and the extent to which each short form was comparable to the full DERS in terms of ability to account for variance in the clinical measures. We will use the acronym “DERS” to refer to both the original DERS and its short forms for the remainder of the paper. When distinguishing between the versions, we will use the term DERS-36 to refer to the original DERS and will use the relevant abbreviations (i.e., DERS-16; DERS-18; and DERS-SF) to refer to each short form.

## Materials and Methods

### Participants

Participants were *N* = 427 adults (59% women; *M* age = 36.00, *SD* = 14.39; 85% White; 3% Black; 3% multiracial; 8% Latino/a) who presented for treatment at an outpatient clinic between September 2014 and January 2017 and diagnosed with one or more *DSM-5* (American Psychiatric Association, 2013) anxiety-related, depressive, bipolar, obsessive-compulsive and related, or trauma- and stressor-related disorders. All participants who met these eligibility criteria and who completed the standard clinical intake were included in analyses. Treatment outcome [Clinical Global Impression (CGI) Scale; Guy, 1976] was available for a substantial subset of participants (*n* = 202) who had completed treatment or had withdrawn from treatment after at least two sessions at the time of the study. Clinical descriptive data are provided in **Table 1**. Comorbidity was common, with 62% having more than one emotional disorder and 34% having diagnoses in more than one class of emotional disorders (e.g., at least one anxiety disorder and at least one depressive disorder). The most common diagnoses (more than 15% of the sample) were OCD (33%), social anxiety disorder (29%), generalized anxiety disorder (28%), panic disorder (21%), persistent depressive disorder (20%), and major depressive disorder (18%). Personality disorders were not systematically assessed.

**Table 1:** Demographic and clinical characteristics.

Age	*M* = 36.00, *SD* = 14.31, *range*: 18–77
Sex	59% Female
Ethnicity	8% Latino/a
Race	85% White
Employment	49% Full time
	14% Student
Education	12% High school or less
	24% Some college or AA
	33% BA/BS or equivalent
	7% Some graduate school
	23% Advanced degree
Intake CGI	*M* = 4.62, *SD* = 0.85, *range*: 2–7
Discharge CGI	*M* = 3.35, *SD* = 1.23, *range*: 1–6
DASS-21	
Stress	*M* = 17.51, *SD* = 9.95, *range*: 0–42
Anxiety	*M* = 12.41, *SD* = 9.41, *range*: 0–42
Depression	*M* = 15.75, *SD* = 12.01, *range*: 0–42
DERS	
Total	*M* = 89.33, *SD* = 22.64, *range*: 37–144
Total without Awareness	*M* = 73.96, *SD* = 21.37, *range*: 30–118
Awareness	*M* = 15.55, SD = 4.92, range: 6–28
Clarity	*M* = 12.01, *SD* = 4.04, *range*: 5–22
Goals	*M* = 15.42, *SD* = 4.215, *range*: 5–21
Impulse	*M* = 12.58, *SD* = 4.97, *range*: 6–25
Non-acceptance	*M* = 14.67, *SD* = 5.92, *range*: 6–24
Strategies	*M* = 19.67, *SD* = 7.31, *range*: 8–33
DSM-5 diagnostic data	
Any anxiety disorder	307 (72%)
Any OC-related disorder	172 (40%)
Any depressive disorder	167 (39%)
Any trauma/stress disorder	22 (5%)
Number of emotional disorders	*M* = 1.99, *SD* = 1.03, *range*: 1–6

*CGI, Clinical Global Impression; DASS-21, Depression, Anxiety, and Stress Scale – 21 Item Version; DERS, Difficulties in Emotion Regulation Scale; OC, obsessive-compulsive*.

### Measures

#### Clinician-Administered Measures

##### Diagnostic interview for anxiety, mood, and obsessive-compulsive and related neuropsychiatric disorders (DIAMOND)

Diagnostic status was established via the DIAMOND (Tolin et al., 2018), a semi-structured diagnostic interview that includes modules for *DSM-5* anxiety, depressive, bipolar and related, obsessive-compulsive and related, and trauma- and stressor-related disorders, and other disorders that are not a focus of the present study (e.g., schizophrenia spectrum disorders; feeding and eating disorders; substance use and addictive disorders). The DIAMOND has generally strong psychometric properties, with interrater reliability coefficients for emotional disorders ranging from κ = 0.62 (very good) to 1.00 (excellent) and test-retest reliability coefficients ranging from κ = 0.59 (good) to 1.00 (excellent) across modules (Tolin et al., 2018). The DIAMOND demonstrates strong convergent validity vis-à-vis self-report measures for the diagnoses included in the present study, as evidenced by significantly higher scores on disorder-specific self-report measures obtained by patients diagnosed with versus not diagnosed with a given emotional disorder (*d* = 0.52–1.22).

##### Clinical global impression (CGI) scale

Clinical severity was established via the CGI (Guy, 1976), a widely used clinician-administered measure that rates global clinical severity on a scale from 1 = *normal, not at all ill* to 7 = *extremely ill*.

#### Self-Report Measures

##### Difficulties in emotion regulation scale (DERS)

The DERS (Gratz and Roemer, 2004) is a 36-item self-report measure of six facets of emotion regulation. Items are rated on a scale of 1 (“*almost never [0–10%]*”) to 5 (“*almost always [91–100%]*”). Higher scores indicate more difficulty in emotion regulation. The psychometric properties of the DERS and its subscales are described throughout the manuscript.

##### Depression anxiety stress scale (DASS-21)

The DASS-21 (Lovibond and Lovibond, 1995) is a 21-item self-report measure that assesses three facets of negative emotion: depression, anxiety, and stress/tension. Each item is rated on a 4-point scale assessing symptom frequency over the past week. Factor analysis supports a three-factor (depression; anxiety; and stress) structure, and the three subscales show good internal consistency, convergent validity, and discriminant validity (Lovibond and Lovibond, 1995; Brown et al., 1997; Antony et al., 1998). A model that includes a higher-order general distress factor represented by the total score also shows good fit (Henry and Crawford, 2005). Subscale scores are created by doubling and summing the items for each subscale. In the current study, the internal consistency was excellent for the DASS depression subscale (α = 0.92) and good for the anxiety (α = 0.84) and stress (α = 0.84) subscales.

### Procedure

Participants completed all self-report measures online as part of the clinic’s standard intake. Participants then met individually with a licensed clinical psychologist or advanced doctoral student trainee to complete the diagnostic interview. All diagnoses and CGIs were confirmed by a licensed clinical psychologist. Diagnosing and treating clinicians were blind to study hypotheses. All patients who met eligibility criteria (i.e., diagnosed with one or more included emotional disorders and completed the DERS) were included. Discharge CGI was available for a subset of *n* = 202 patients who had completed or withdrawn from treatment at the time of the study and who had attended at least two treatment sessions. Treatment consisted of a naturalistic course of cognitive-behavioral therapy in an outpatient setting. Discharge CGI was determined by the patient’s primary clinician and was confirmed by the licensed supervisor in the case of trainee clinicians.

### Analytic Plan

To establish the factor structure of the DERS in treatment-seeking adults with emotional disorders, we tested three previously identified factor structures using confirmatory factor analysis (CFA) in MPlus 7.0 (Muthén and Muthén, 1998–2017). These included the original six-factor structure (Gratz and Roemer, 2004; Weinberg and Klonsky, 2009; Neumann et al., 2010), a five-factor structure that excludes the Awareness items (Bardeen et al., 2012; Fowler et al., 2014), and a bifactor model with one general factor and five specific factors (again, with Awareness items excluded; Osborne et al., 2017). The WLSMV estimator was used for all analyses to account for the categorical response scales. To assess incremental utility, we tested a structural equation model (SEM) in which the best-fitting DERS factor structure (identified in the previous step) was tested as a predictor of a latent “clinical severity” variable comprised of clinician-rated CGI and the three DASS subscales. This approach allowed us to assess the unique incremental contribution of each DERS latent factor (i.e., the contribution after all other latent factors were controlled) to clinical severity. To test predictive utility, we expanded the SEM to include discharge CGI, which allowed us to test the unique contributions of each DERS factor to clinical outcome beyond variance explained by baseline clinical severity. We elected to use discharge CGI rather than clinician-rated improvement for ease of interpretation (i.e., because improvement is necessarily confounded with clinical severity at intake, which was already statistically controlled within the model). Following guidelines provided by MacCallum et al. (1996), we established that we had >99% power to detect a model with acceptable fit (RMSEA < 0.06) for the two baseline models and >87% power to detect a model with acceptable fit for the smaller treatment outcome model. After establishing the best-fitting baseline model for the DERS-36, we assessed the extent to which each short form conformed to that model using confirmatory structural equation modeling. Finally, we assessed the psychometric properties of the DERS short forms by examining the internal consistency of each subscale and its concordance with the corresponding DERS-36 subscale. We also ran a series of hierarchical regressions to test the extent to which each short form fully accounted for DERS-related variance in clinical severity. For each short form, its subscale scores were entered on the first step and the DERS-36 subscales were entered on the second step. This approach allowed us to determine whether the DERS-36 captured additional variance in each outcome beyond the variance explained by short form. Missing data represented less than 1% of all observations and was addressed using pairwise deletion.

## Results

### Preliminary Analyses

Independent *t*-tests and correlation analyses were conducted to examine the relationship of each subscale to gender and age, respectively. Women scored higher than men on Impulse [*t*(407) = 2.33, *p* = 0.020] and marginally higher on Non-acceptance [*t*(415) = 1.86, *p* = 0.064]. No differences were observed for the total score or the other subscales. Age showed small but significant negative associations with the DERS total score (*r* = -0.16, *p* = 0.002) and all subscales (*r* ≥ -0.12, *p* ≤ 0.018) except Awareness (*r* = -0.004, *p* = 0.240). Bivariate correlations between the DERS and other clinical measures are presented in **Table 2**.

**Table 2:** Bivariate correlations.

	2	3	4	5	6	7	8	9	10	11	12	13
(1) Awareness	0.42^∗∗^	-0.01	0.09^†^	0.10^†^	0.04	0.35^∗∗^	0.14^∗∗^	0.06	0.05	0.02	0.12^∗^	0.15^∗^
(2) Clarity	–	0.34^∗∗^	0.44^∗∗^	0.39^∗∗^	0.44^∗∗^	0.67^∗∗^	0.62^∗∗^	0.40^∗∗^	0.43^∗∗^	0.43^∗∗^	0.30^∗∗^	0.18^∗^
(3) Goals	–	–	0.59^∗∗^	0.45^∗∗^	0.68^∗∗^	0.72^∗∗^	0.76^∗∗^	0.48^∗∗^	0.29^∗∗^	0.48^∗∗^	0.31^∗∗^	0.26^∗∗^
(4) Impulse	–	–	–	0.57^∗∗^	0.72^∗∗^	0.81^∗∗^	0.84^∗∗^	0.52^∗∗^	0.41^∗∗^	0.53^∗∗^	0.30^∗∗^	0.14^†^
(5) Non-acceptance	–	–	–	–	0.67^∗∗^	0.79^∗∗^	0.81^∗∗^	0.42^∗∗^	0.42^∗∗^	0.47^∗∗^	0.19^∗∗^	0.13^†^
(6) Strategies	–	–	–	–	–	0.88^∗∗^	0.92^∗∗^	0.65^∗∗^	0.44^∗∗^	0.55^∗∗^	0.34^∗∗^	0.18^∗^
(7) DERS total	–	–	–	–	–	–	0.98^∗∗^	0.61^∗∗^	0.49^∗∗^	0.59^∗∗^	0.38^∗∗^	0.24^∗∗^
(8) DERS total without Awareness	–	–	–	–	–	–	–	0.63^∗∗^	0.50^∗∗^	0.62^∗∗^	0.36^∗∗^	0.22^∗∗^
(9) DASS-depression	–	–	–	–	–	–	–	–	0.56^∗∗^	0.60^∗∗^	0.45^∗∗^	0.30^∗∗^
(10) DASS-anxiety	–	–	–	–	–	–	–	–	–	0.63^∗∗^	0.36^∗∗^	0.10
(11) DASS-stress	–	–	–	–	–	–	–	–	–	–	0.38^∗∗^	0.16^∗^
(12) Intake CGI	–	–	–	–	–	–	–	–	–	–	–	0.55^∗∗^
(13) Discharge CGI	–	–	–	–	–	–	–	–	–	–	–	–

^†^*p < 0.10; ^∗^*p* < 0.05; ^∗∗^*p* < 0.01*.

### Factor Structure of the DERS-36

All factor analyses and structural models were tested using MPlus 7.0 with an estimator appropriate for categorical response scales (WLSMV). We first attempted to fit the original six-factor model (Gratz and Roemer, 2004). Fit was poor, χ^2^(579) = 2084.31, *p* < 0.001, RMSEA = 0.08, CFI = 0.94, TLI = 0.93. Fit for a five-factor model that excluded the Awareness items was better, χ^2^(395) = 1478.74, *p* < 0.001, RMSEA = 0.08, CFI = 0.95, TLI = 0.95, but was still unacceptable by some commonly used standards (e.g., acceptable RMSEA < 0.06 or 0.07; Hu and Bentler, 1999; Steiger, 2007).

**FIGURE 1:**
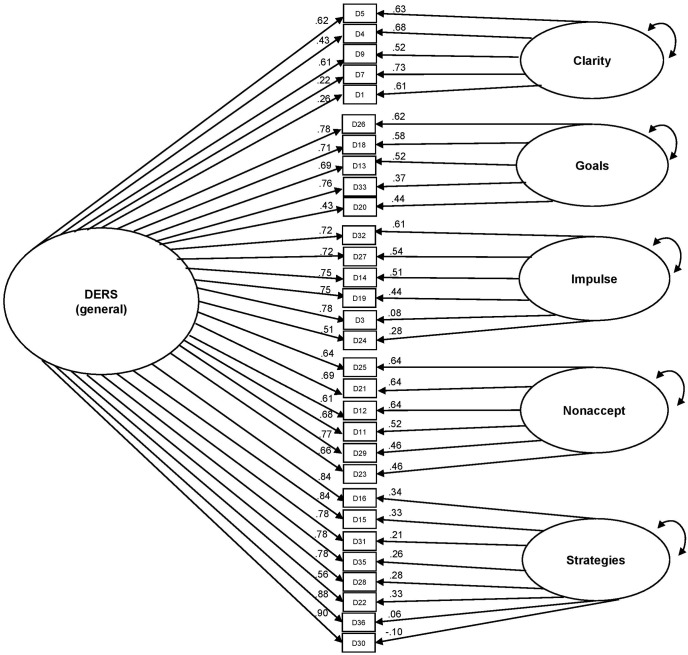
Bifactor model of the DERS, excluding Awareness items.

Finally, we tested a bifactor model consisting of one general factor upon which all items were permitted to load (Awareness items were excluded) and five uncorrelated specific factors upon which only the items that comprised each subscale were permitted to load (see **Figure 1**). Model fit was acceptable, χ^2^(375) = 1016.70; RMSEA = 0.06; CFI = 0.97; TLI = 0.97. All items loaded significantly on the general factor and all loadings were >0.40, except for two items from the Clarity subscale (items 1 [“I am clear about my feelings”] and 7 [“I know exactly how I am feeling”]; standardized loading range: 0.26–0.90). All items also loaded significantly on their specific factor (beyond variance explained by the general factor) with the exception of two items from the Strategies subscale (items 30 [“When I’m upset, I start to feel very bad about myself”] and 36 [“When I’m upset, my emotions feel overwhelming”]).

### Incremental Utility

**FIGURE 2:**
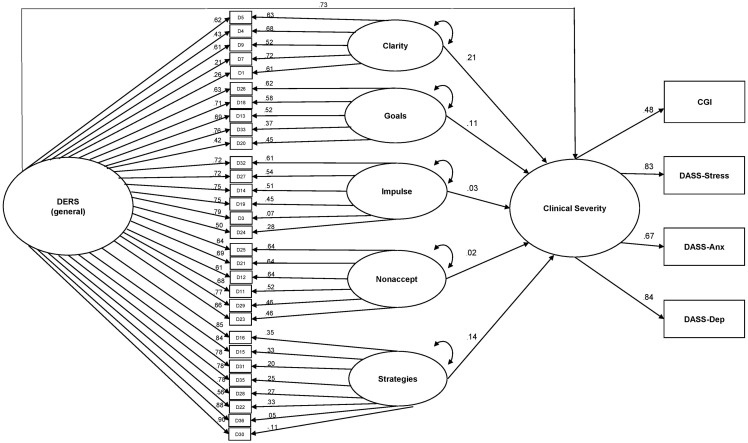
Contributions of the DERS general and specific factors to predicting clinical severity.

After identifying the six-factor (1 general and 5 specific) bifactor solution as providing the best fit to the data, we compared two competing structural equation models to examine the extent to which the specific factors could explain variance in clinical severity beyond variance accounted for by the general factor. Model comparison significance testing was conducted using the DIFFTEST command, which is appropriate for models that use the WLSMV estimator (Muthén and Muthén, 1998–2017). Both models included a clinical severity latent variable (observed variables were CGI and the three DASS subscales) which was predicted by the DERS bifactor model (excluding Awareness). The first model allowed only the general factor from the bifactor model to predict clinical severity; paths were not included for the subscale scores. Model fit was acceptable, χ^2^(496) = 1257.08, *p* < 0.001; RMSEA = 0.06; CFI = 0.96; TLI = 0.96. In the second model (see **Figure 2**), paths were added for the five specific factors. Model fit was significantly improved by this modification, Δχ^2^(5) = 28.69, *p* < 0.001; model χ^2^(491) = 1231.48, *p* < 0.001, RMSEA = 0.06, CFI = 0.97, TLI = 0.96. Significant unique paths were observed for the Strategies (0.14, *p* = 0.012), Goals (0.11, *p* = 0.022), and Clarity (0.21, *p* < 0.001) specific factors.

### Predictive Utility

Discharge CGI was available for *n* = 202 participants. Participants who did not have discharge data (i.e., who were still in treatment at the time of the study or who withdrew from treatment before their second session) reported significantly greater anxiety, *t*(409.76) = 2.75, *p* = 0.006, and depression, *t*(414.31) = 2.16, *p* = 0.031, at intake compared to participants with discharge data. However, the groups did not differ in age (*p* = 0.984), gender (*p* = 0.108), CGI (*p* = 0.660), or DERS total or subscale scores (all *p* ≥ 0.274).

**FIGURE 3:**
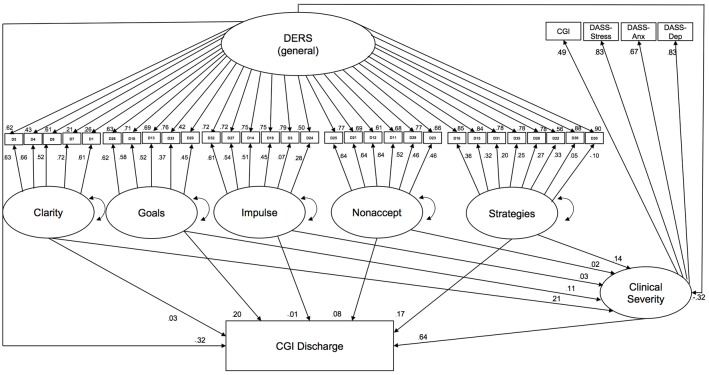
Contribution of the DERS common and specific factors to predicting clinical severity at discharge.

To assess the extent to which the DERS and its subscales incrementally predicted treatment outcome, we expanded the structural model described above (see Incremental Validity) by adding discharge CGI as an outcome (**Figure 3**). The final model examined the extent to which the general factor and each subscale predicted severity at discharge, controlling for severity at intake. Model fit was acceptable χ^2^(518) = 1337.06, *p* < 0.001; RMSEA = 0.06; CFI = 0.96; TLI = 0.96. The latent general factor and the Goals specific factor each explained significant incremental variance in discharge CGI. The Goals specific factor was positively associated with CGI at discharge (after controlling for other variables). Surprisingly, the general factor was negatively associated with discharge CGI after controlling for the other variables, suggesting that poorer emotion regulation (as assessed by the DERS) predicted a better clinical outcome after other factors were controlled. These findings should be interpreted with caution, since the smaller number of participants with discharge CGI data reduced statistical power for this analysis.

### Psychometric Properties of Short Forms

**Table 3** provides the internal consistency of each short form subscale (Cronbach’s alpha) and their association with the corresponding original DERS subscale (Pearson correlation). The short forms generally showed adequate reliability and good concordance with the original subscale, with Awareness performing somewhat worse, as expected.

**Table 3:** Internal consistency of short form subscale and total scores and correlation with original (36-item) DERS scores.

Original DERS	DERS-18	DERS-16	DERS-SF
Awareness (α = 0.82)	0.92^∗∗^ (0.79)	–	0.92^∗∗^ (0.75)
Clarity (α = 0.82)	0.92^∗∗^ (0.81)	0.86^∗∗^ (0.81)	0.92^∗∗^ (0.81)
Goals (α = 0.86)	0.95^∗∗^ (0.87)	0.96^∗∗^ (0.83)	0.95^∗∗^ (0.87)
Impulse (α = 0.88)	0.93^∗∗^ (0.89)	0.95^∗∗^ (0.87)	0.93^∗∗^ (0.89)
Non-acceptance (α = 0.92)	0.96^∗∗^ (0.87)	0.96^∗∗^ (0.86)	0.98^∗∗^ (0.87)
Strategies (α = 0.92)	0.94^∗∗^ (0.83)	0.98^∗∗^ (0.89)	0.95^∗∗^ (0.83)
Total (α = 0.94)	0.97^∗∗^ (0.89)	0.94^∗∗^ (0.94)	0.97^∗∗^ (0.89)
Total without awareness (α = 0.95)	0.98^∗∗^ (0.92)	0.98^∗∗^ (0.94)	0.98^∗∗^ (0.92)

*^∗∗^p < 0.001. Cronbach’s alpha in parentheses; DERS, Difficulties in Emotion Regulation Scale*.

Confirmatory bifactor modeling was conducted for each short form to examine the extent to which each form replicated the bifactor structure of the DERS-36. Awareness subscales were excluded from analyses. The bifactor structure provided an acceptable fit for all three short forms (DERS-16: χ^2^(79) = 191.93, *p* < 0.001, RMSEA = 0.06, CFI = 0.99; DERS-18: χ^2^(66) = 98.78, *p* = 0.006, RMSEA = 0.03, CFI = 1.00; DERS-SF: χ^2^(66) = 89.15, *p* = 0.030, RMSEA = 0.03, CFI = 1.00).

To examine the incremental utility and reliability of each short form vis-à-vis the DERS-36, we conducted a series of hierarchical regression analyses. Age and gender were entered on Step 1. CGI was also entered on Step 1 for the DASS analyses. All short form subscales excluding Awareness were entered on Step 2, and all DERS-36 subscales excluding Awareness were entered on Step 3 (see **Table 4**). This approach allowed us to examine the extent to which the short forms compare to the full DERS-36 in terms of explaining variance in the clinical outcomes. To conserve statistical power, we examined only the omnibus *F*-tests and *R*-squared change for each short form rather than evaluating each of the short forms’ subscales separately.

**Table 4:** Incremental utility of the DERS-16, DERS-18, and DERS-SF.

	CGI		Anxiety		Depression		Stress	
All models	Δ*R*^2^	*P*	Δ*R*^2^	*P*	Δ*R*^2^	*P*	Δ*R*^2^	*P*
Step 1: Age, gender, and CGI^a^	0.00	0.704	0.15	<0.001	0.20	<0.001	0.16	<0.001
DERS-16								
Step 2: DERS-16 subscales	0.14	<0.001	0.19	<0.001	0.29	<0.001	0.27	<0.001
Step 3: DERS-36 subscales	0.03	0.010	0.01	0.167	0.03	<0.001	0.00	0.804
DERS-18								
Step 2: DERS-18 subscales	0.15	<0.001	0.20	<0.001	0.31	<0.001	0.27	<0.001
Step 3: DERS-36 subscales	0.03	0.029	0.02	0.077	0.02	0.003	0.01	0.094
DERS-SF								
Step 2: DERS-SF subscales	0.16	<0.001	0.20	<0.001	0.31	<0.001	0.27	<0.001
Step 3: DERS-36 subscales	0.01	0.422	0.03	0.009	0.03	0.001	0.02	0.029

*DERS, Difficulties in Emotion Regulation Scale; CGI, Clinical Global Impression; ^*a*^analyses with CGI as the dependent variable include only age on the first step. All analyses exclude the Awareness subscale(s)*.

The overall pattern of results was similar across the short forms, each of which explained 14–16% of the variance in CGI after controlling for age and gender and 19–20% of the variance in anxiety, 29–31% of the variance in depression, and 27% of the variance in stress after controlling for age, gender, and CGI (**Table 4**). The DERS-36 accounted for a small but significant additional portion of the variance (2–3%) in depression for all three short forms. The DERS-36 also explained an additional small but significant amount of variance (2–3%) in CGI for the DERS-16 and the DERS-18, and 2–3% of the variance in anxiety and stress for the DERS-SF. That is, the DERS-16 and DERS-18 were not inferior to the DERS-36 in their ability to account for variance in anxiety and stress, and the DERS-SF was not inferior to the DERS-36 in its ability to account for variance in CGI.

## Discussion

The present study aimed to characterize the psychometric properties of the DERS in a large transdiagnostic sample of treatment-seeking adults with emotional disorders. Overall, the original DERS (DERS-36; Gratz and Roemer, 2004) and its three newly developed short forms showed psychometric properties that ranged from adequate to good across a range of indices. The factor analytic results suggested that a bifactor solution with one general factor and five specific factors (Awareness excluded) provided the best fit to the data. These findings were bolstered by our SEM findings, which demonstrated specific contributions of three of the five subscales to explaining variance in clinical severity beyond variance accounted for by the general factor. There was also some preliminary support for the utility of the DERS for predicting treatment outcome following outpatient CBT.

Consistent with findings from a wide array of existing studies (e.g., Osborne et al., 2017), the Awareness subscale showed relatively poor psychometric properties in both the DERS-36 and its short forms. Internal consistency was poor (α < 0.80) and internal consistency for the total score was reliably improved by the exclusion of those items. Convergent validity for the total scores of the short forms (vis-à-vis the DERS-36) was also improved when the Awareness items were not included in the total. The consistency of these findings, both in the present study and in the extant literature, leads us to conclude that the DERS as a whole is psychometrically stronger when the Awareness subscale is excluded. One possible reason for this is that the Awareness subscale assesses a different construct. Whereas the other DERS subscales aim to assess how an individual *reacts* to emotions, the Awareness subscale aims to assess whether or not an individual *notices* emotions. Emotional awareness may be a necessary but insufficient criterion for emotion regulation, but it does not appear to be the same construct. We therefore recommend that the Awareness subscale be excluded from future research using the DERS, or at a minimum excluded from the total score interpreted separately and with caution.

In contrast to the Awareness findings, the other subscales performed reasonably well in terms of internal consistency, convergent validity (in the case of the short forms), and incremental and predictive utility. With respect to incremental utility, the most compelling support comes from our factor analytic and SEM results. As described above, compared to the original six-factor solution (Gratz and Roemer, 2004) and the popular five-factor solution with Awareness excluded (Bardeen et al., 2012; Fowler et al., 2014; Osborne et al., 2017), a bifactor solution (comprised of one general “DERS” factor and five specific factors representing the variance that is unique to each subscale) best fit the data. This finding provides support for the specificity of at least some of the factors and suggest that the present findings are not attributable to general distress or common-method variance (i.e., because variance due to these factors would be subsumed under the general factor). The presence of a general factor suggests the possibility of a latent “emotion regulation” construct underlying the DERS, although this possibility will need to be explored in future research aimed at testing the convergent and discriminant validity of the general factor vis-à-vis other measures of emotion regulation. The significant and generally strong loadings of each DERS item on the general factor also suggests that the items tap into the same general construct and provides support for the validity of a total score as a representation of this construct.

When clinical severity was added to the model, a solution that permitted both the general and specific factors to predict variance provided a superior fit to the data compared to a model where only the general factor was allowed to predict variance, providing further support for the bifactor model. The Strategies, Goals, and Clarity specific factors in particular contributed significant unique variance to explaining clinical severity even after accounting for the general latent factor and the unique contributions of each other factor. Taken together, these findings suggest that useful information may be gleaned both from the total score and from the subscale scores. In a third model that included treatment outcome data, the general factor and the Goals factor each predicted outcome even after clinical severity at intake and the other specific factors were controlled. Notably, the DERS general latent factor was associated with better treatment outcome (i.e., lower CGI at discharge after controlling for baseline clinical severity), while the Goals factor was associated with poorer outcome.

The finding that poorer baseline emotion regulation (as represented by the general factor) was associated with better CBT outcome (i.e., that participants with poorer emotion regulation at baseline improved more following a naturalistic course of CBT) was unexpected. One possible explanation is that participants who began the study with poorer overall emotion regulation skills were able to benefit more from the specific strategies taught in CBT, many of which aim to remediate these deficits. As such, these participants may have had more opportunity to improve relative to baseline compared to participants who had already mastered these skills. Additional research using a prospective design with multiple assessments will be needed to assess this potential mediational pathway. The finding that poorer perceived ability to engage in goal-directed cognition and behavior when distressed (as represented by the Goals factor) predicted poorer outcome was less surprising. One likely explanation is that individuals who struggle with cognitive and behavioral control when distressed may be less likely to attend sessions or complete CBT homework, which would in turn lead to poorer outcome. If this proposed pathway is true, it would suggest that patients may benefit from specific training in cognitive control or self-regulation of behavior early in treatment. Future research that includes assessments of these potential mediators is needed to test this potentially important relationship. This research should also clarify which facets of the Goals construct (i.e., cognitive control versus self-regulation of behavior) are most strongly predictive of treatment outcome. Because the DERS is a self-report measure, future research should also use both self-report and behavioral assessments to establish whether actual cognitive or self-control, perceived cognitive- or self-control, or both, best account for the observed relationships.

With respect to the DERS short forms, the three we examined (DERS-16; DERS-18; and DERS-SF) generally showed strong concordance with the original DERS-36. Internal consistency was generally fair-to-good, and was reliably above 0.80 for all subscales except Awareness. A bifactor model following the general and specific factor structure observed for the DERS-36 (i.e., one general factor and five specific factors corresponding to each subscale excluding Awareness) provided a good fit for each short form, suggesting good concordance in factor structure. Concordance of the short form subscales with the corresponding DERS-36 subscales was also high; all were greater than *r* = 0.86 and most were greater than *r* = 0.90. Nevertheless, the DERS-36 explained a small (1–3%) but significant percentage of variance in several clinical variables beyond variance explained by each short form, suggesting that some potentially useful information may be lost with the reduction of scale length. Whether the benefits of reduced patient or participant burden outweighs this relatively minor loss of information is a decision for individual clinicians and researchers depending on their priorities (i.e., comprehensiveness versus reduced participant/patient burden). In our opinion, the present results suggest that these three short forms may be acceptable alternatives to the DERS in many clinical and treatment scenarios. We do not believe there is sufficient evidence to suggest that any short form is psychometrically superior to the others.

These findings should be interpreted in light of several strengths and limitations. One notable strength is our use of a relatively large sample of treatment-seeking adults with emotional disorders. This sample provides an ecologically valid context for assessing the psychometric properties of the DERS, which is based on a clinically derived model of emotion regulation and often used in similar clinical contexts. A related strength is the inclusion of a prospective study arm. This prospective component is admittedly limited in several ways; we were not able to distinguish between treatment completers and treatment drop-outs, and only one index of clinical severity (clinician-rated CGI) was available. Nevertheless, the present findings provide some of the first evidence to support the predictive validity of the DERS in a cognitive-behavioral treatment context.

One important limitation is that the present findings do not address the convergent and construct validity of the DERS vis-à-vis other measures of emotion regulation ability. However, the finding that several DERS subscales incrementally accounted for variance across several indices of emotion-related psychopathology (anxiety; depression; and stress) is at least consistent with the notion of construct validity, particularly when the findings are situated within the framework proposed by Gratz and Roemer (2004), which argues for specific contributions of each facet to emotion-related psychopathology. To disentangle difficult questions about validity, future research on the DERS should include other validated self-report measures of emotion regulation as well as measures of constructs that are represented within the DERS but which are not defined as “emotion regulation” *per se* within the broader emotion regulation literature (e.g., Gross, 1998; Sheppes et al., 2015), such as alexithymia (Clarity), self-control (Impulse), and cognitive control or self-control (Goals). Future research should also carefully examine the Strategies subscale, which performed well in the present study but includes depression-related content (e.g., “When I’m upset, I believe that I’ll end up feeling very depressed;” “When I’m upset, I start to feel very bad about myself”), which may inflate validity estimates. Also critical for future validation work is the inclusion of non-self-report (e.g., behavioral, physiological, or neurobiological) indices of emotion regulation ability. These convergent sources of data may be useful for addressing general problems related to self-report data (e.g., demand characteristics), but also for a more specific problem related to the assessment of emotion regulation, which is that the ability to experience a desired emotional end-state (i.e., to feel better) relies not just on emotion regulation ability, but on the initial intensity of the emotion to be regulated. Put another way, the same “dose” of emotion regulation might result in mild distress for an individual whose emotion was moderately intense prior to regulation, but moderate distress for an individual whose emotion was very intense prior to regulation (e.g., Lewis et al., 2010).

To our knowledge, this study is the first to examine the general and specific concurrent and predictive validity of the DERS and its subscales using an SEM framework. Within that framework, we replicated and extended Osborne et al.’s (2017) finding that a bifactor solution excluding the Awareness subscale provides a good fit for the data. Moreover, our data suggest that several specific factors predict clinical severity and, to a lesser extent, treatment outcome, even after variance explained by the general underlying factor is controlled. The short forms generally performed similarly well despite a slight loss of predictive utility (1–3% of the variance) for explaining clinical severity. These findings suggest that the use of a short form to reduce participant burden would likely be acceptable in most clinical and research situations. Taken together, these findings suggest that after excluding the Awareness subscale, the DERS demonstrates good internal consistency, a robust bifactor structure that is consistent with the commonly used subscales, and good evidence of incremental and predictive utility. These findings also suggest that the DERS may have clinical utility both as an index of emotion dysregulation and as a potential prognostic indicator. In particular, clinicians should pay special attention to participants’ reports of their ability to engage in goal-directed cognition and behavior when distressed (i.e., Goals score) as this appears to have unique incremental predictive power beyond other subscales and the general factor. Future research should include alternative measures of emotion regulation (including behavioral or physiological measures) and related constructs (cognitive control; alexithymia) to further evaluate validity of the DERS as a measure of emotion regulation *per se* and to clarify the mechanisms by which the various facets of emotion dysregulation assessed by the DERS relate to clinical severity, functional impairment, and distress. Future research should also clarify the mechanisms by which the DERS predicts treatment outcome, with the ultimate goal of deriving specific implications for treatment.

## Ethics Statement

The study was carried out in accordance with the recommendations of the Hartford HealthCare (HHC) Institutional Review Board. A waiver of informed consent was obtained for the study. The protocol was approved by the Hartford HealthCare (HHC) Institutional Review Board.

## Author Contributions

LH performed the statistical analyses and wrote the first draft of the manuscript. All authors contributed to study conception and design and approved the final version of the manuscript.

## Conflict of Interest Statement

The authors declare that the research was conducted in the absence of any commercial or financial relationships that could be construed as a potential conflict of interest.
